# The effects of SARS-CoV-2 on bone homeostasis

**DOI:** 10.3389/fendo.2026.1897220

**Published:** 2026-08-03

**Authors:** M. Victoria Delpino, Jorge Quarleri

**Affiliations:** Instituto de Investigaciones Biomédicas en Retrovirus y Sida (INBIRS), Facultad de Ciencias Médicas, Universidad de Buenos Aires-Consejo de Investigaciones Científicas y Técnicas (CONICET), Buenos Aires, Argentina

**Keywords:** bone, osteoblast (OB), osteoclast (OC), osteoporosis, SARS-CoV-2

## Abstract

Accumulating evidence suggests that SARS-CoV-2 is associated with disruptions in skeletal homeostasis through both direct viral effects on bone-remodeling cells and indirect inflammatory mechanisms, contributing to bone loss and increased skeletal fragility. The maintenance of healthy bone structure depends on the coordinated activities of bone-resorbing osteoclasts and bone-forming osteoblasts. Recent studies demonstrate that differentiated human osteoblasts support productive SARS-CoV-2 infection, whereas MSC and osteoclast precursors undergo abortive infection. These interactions impair osteoblast differentiation, increase IL-6 and receptor activator of nuclear factor κB Ligand (RANKL) expression, and promote osteoclastogenesis, shifting bone remodeling toward pathological resorption. Additional virus-associated mediators, including Spike protein, ORF8, and miR-4485-3p, further amplify inflammatory and pro-osteoclastogenic signaling. Clinical studies report associations between acute and persistent reductions in bone mineral density, increased vertebral fracture prevalence, and alterations in musculoskeletal (MSK) imaging biomarkers associated with worse COVID-19 outcomes, while animal models confirm osteoclast-driven trabecular bone loss. This review integrates emerging evidence of productive and abortive SARS-CoV-2 infection of human bone cells with recent clinical and experimental findings, providing an updated osteoimmunological perspective on COVID-19-associated bone remodeling. Prospective longitudinal studies and clinical trials are needed to determine the long-term skeletal consequences of COVID-19 and evaluate targeted strategies to prevent infection-associated bone loss.

## Introduction

The World Health Organization announced in May 2023 that COVID-19 was no longer classified as a public health emergency of international concern (https://www.who.int/news/item/05-05-2023-statement-on-the-fifteenth-meeting-of-the-international-health-regulations-(2005)-emergency-committee-regarding-the-coronavirus-disease-(covid-19)-pandemic). Despite this milestone, accumulating evidence indicates that infection with severe acute respiratory syndrome coronavirus 2 (SARS-CoV-2) can lead to persistent alterations across multiple physiological systems (1–5). SARS-CoV-2 is an RNA virus whose pathogenic process begins with entry into susceptible host cells. Viral entry is mediated by the surface spike (S) protein, which binds to angiotensin-converting enzyme 2 (ACE2) receptors expressed in a wide range of tissues, including the lungs, kidneys, gastrointestinal tract, heart, liver, bone, and vascular endothelium (6–9).

Recent studies have demonstrated the presence of SARS-CoV-2 RNA and viral protein components in tissues beyond the respiratory tract, supporting the concept of multi-organ dissemination and long-term viral persistence, particularly among immunocompromised patients (10–15). A subset of COVID-19 survivors develops prolonged symptoms collectively referred to as long COVID. Recent reports have shown that SARS-CoV-2 antigens can persist in circulation for months after acute infection. Circulating spike, S1, or nucleocapsid proteins have been detected up to 14 months post-infection and are more frequently observed in individuals with post-acute sequelae of COVID-19, suggesting a potential link between persistent viral antigens and ongoing symptoms (16).

In addition, intra-host viral diversity across different organs suggests ongoing viral adaptation, which may contribute to sustained biological effects even after resolution of acute infection (17). Together, these findings reinforce the notion that SARS-CoV-2 infection can have long-lasting systemic consequences with important implications for tissue and organ homeostasis.

Although the respiratory manifestations of COVID-19 are well characterized, its extrapulmonary effects, particularly on the skeletal system, remain incompletely understood. Emerging clinical and experimental evidence suggest and association between SARS-CoV-2 infection and impaired bone health, reduced bone mineral density, increased vertebral fracture risk, and alterations in bone remodeling (4, 5, 18).

While previous reviews have primarily emphasized indirect mechanisms, including systemic inflammation, corticosteroid therapy, vitamin D deficiency, and reduced mobility (4, 5, 18–21), recent studies have demonstrated that SARS-CoV-2 can directly interact with cells of the bone-remodeling lineage (7, 22–29). These findings provide new insights into potential mechanisms underlying COVID-19-associated bone loss. Although recent reviews have acknowledged some of these emerging findings, they have not comprehensively integrated the rapidly expanding evidence on direct SARS-CoV-2 interactions with bone-remodeling cells, the roles of viral proteins and virus-induced microRNAs, and their implications for osteoimmunological dysregulation and skeletal health.

In this minireview, we summarize current clinical evidence and integrate recently published mechanistic studies, including our own findings demonstrating productive infection of differentiated human osteoblasts and abortive infection of MSC and osteoclast precursors by SARS-CoV-2. We further discuss the roles of viral proteins, virus-induced microRNAs, and osteoimmunological pathways in disrupting bone homeostasis. By integrating these recent advances, this review provides an updated mechanistic framework that builds upon previous reviews by highlighting how direct viral effects, osteoimmunological pathways, and molecular mechanisms converge to disrupt bone homeostasis and by discussing their implications for post-COVID skeletal monitoring, osteoporosis risk assessment, optimization of vitamin D and calcium status, and targeted anti-resorptive or anabolic therapeutic interventions (4, 5, 19–21).

This narrative mini-review was based on a literature search conducted in PubMed/MEDLINE, Scopus, and Google Scholar for articles published from January 2020 through April 2026. Search terms included combinations of “COVID-19”, “SARS-CoV-2”, “bone”, “bone remodeling”, “osteoblast”, “osteoclast”, “osteoporosis”, “bone mineral density,” “fracture”, “osteoimmunology”, and “long COVID”. Priority was given to original experimental studies, clinical investigations, systematic reviews, and meta-analyses relevant to the effects of SARS-CoV-2 on skeletal biology and bone health. Additional relevant publications were identified through the reference lists of selected articles.

## Clinical evidence of the SARS-CoV-2 impact on bone

### Bone mineral density changes after COVID-19

Osteoporosis is a multifactorial metabolic bone disease characterized by low bone mass and abnormal bone microarchitecture (30); studies report that patients with this condition are more likely to acquire SARS-CoV-2, can experience worsening bone loss after COVID-19, and that some COVID-19 cases develop osteoporosis as a complication (31).

Vitamin D deficiency, which is common in severe COVID-19 and associated with increased mortality, impairs bone health, calcium homeostasis, and immune function, while also modulating the renin–angiotensin system (RAS) used by SARS-CoV-2 for cellular entry (32–35).

Early studies described an osteometabolic pattern in COVID-19 patients—acute hypocalcemia, low vitamin D with an inadequate parathyroid hormone (PTH) response, and more vertebral fractures, which, along with multiple reports of post-COVID secondary osteoporosis, this underscores the disruptive impact of the virus on bone metabolism (36).

Interpretation of low 25-hydroxyvitamin D concentrations in COVID-19 requires caution because reverse causality remains a major concern: patients with severe illness, obesity, advanced age, chronic disease, or prolonged hospitalization are more likely to be vitamin D deficient before infection and may also exhibit acute reductions in circulating 25-hydroxyvitamin D during systemic inflammation (37, 38). Thus, low vitamin D levels may represent both a marker of poor baseline health and a potential contributor to adverse skeletal outcomes. For patients undergoing post-COVID bone health assessment, measurement of serum calcium, phosphate, albumin-adjusted calcium, 25-hydroxyvitamin D, and PTH is reasonable, particularly in those with severe disease, prolonged corticosteroid exposure, or suspected osteoporosis. Repeating testing after recovery may help distinguish transient illness-related changes from persistent endocrine abnormalities (19, 32–36).

Overall, the evidence indicates that SARS-CoV-2 acute infection is associated with an increased risk of adverse changes in bone mineral density (BMD) among affected individuals (39–43). A detrimental effect of COVID-19 on BMD was already evident during the acute phase of infection in hospitalized patients, with a noticeable reduction in bone density observed during short-term follow-up. Additionally, the proportion of patients meeting criteria for osteoporosis increased markedly after hospitalization. Importantly, greater bone loss was associated with longer hospital stays, while no substantial differences were reported between men and women (40).

The absence of statistically significant sex differences in some hospitalized cohorts should be interpreted cautiously because most studies were not powered to evaluate menopausal status, circulating estradiol concentrations, or use of menopausal hormone therapy. Estrogen deficiency is a major determinant of postmenopausal bone loss through increased RANKL expression, reduced osteoprotegerin (OPG) production, and enhanced osteoclast survival (44–46). Consequently, SARS-CoV-2-associated inflammatory signaling may act synergistically with pre-existing estrogen deficiency to accelerate bone resorption and increase skeletal fragility in postmenopausal women. Future longitudinal studies should therefore stratify participants by menopausal status, sex steroid exposure, and hormone therapy use when assessing post-COVID skeletal outcomes.

Early post-infection assessments also revealed a high prevalence of osteoporosis among patients hospitalized for COVID-19 who presented with MSK complaints, when compared with non-infected controls (41). These skeletal alterations were not limited to the acute phase. Individuals with osteopenia or osteoporosis who were receiving treatment demonstrated further reductions in BMD at both the lumbar spine and femur relative to baseline. Patients who experienced more severe forms of COVID-19 consistently showed the greatest declines in bone density, with losses exceeding those observed in individuals with milder disease courses (39). Similarly, individuals evaluated several months after recovery continued to exhibit lower BMD than control populations, suggesting that skeletal alterations may persist beyond the acute illness (42).

Because most of these studies involved hospitalized patients, an important confounding factor is prolonged immobilization. Bone loss observed after severe COVID-19 may not be attributable exclusively to SARS-CoV-2 infection. Prolonged hospitalization, intensive care unit admission, mechanical ventilation, physical inactivity, and the associated hypercatabolic state are well-established causes of disuse osteoporosis and can independently accelerate bone resorption (41). Experimental bed-rest studies have demonstrated bone mineral density losses of approximately 0.5–1.0% per month at weight-bearing skeletal sites, whereas patients with spinal cord injury may experience substantially greater and more rapid declines during the first months after injury, highlighting the profound skeletal consequences of mechanical unloading (47, 48). Distinguishing virus-specific effects from immobilization-related bone loss will require prospective studies comparing patients with COVID-19 and non-COVID critically ill populations with similar degrees of immobilization and treatment exposures. Current evidence is insufficient to determine the relative contribution of these factors. Nevertheless, early mobilization and progressive weight-bearing rehabilitation remain important strategies to help minimize bone loss during recovery.

Individuals evaluated several months after recovery continued to exhibit lower bone density compared with control populations, suggesting that the skeletal effects of COVID-19 may extend beyond the acute illness phase (42).

Corticosteroid therapy emerged as an important contributing factor to bone loss. Patients treated with corticosteroids experienced significantly greater reductions in lumbar and femoral BMD, particularly those exposed to higher cumulative doses or longer treatment durations. These findings support a dose- and time-dependent association between corticosteroid use during COVID-19 management and subsequent bone loss (39, 40).

In addition to the effects observed by DXA, several studies have explored whether routinely acquired chest CT scans can provide complementary information on skeletal health. In all the following studies, chest computed tomography (CT) was acquired at hospital admission as part of the initial clinical assessment of patients with COVID-19, and the imaging-derived biomarkers were therefore measured on baseline scans obtained during the acute phase of illness. Opportunistic analysis of these routine admission CT scans consistently identified MSK and metabolic indicators, most notably vertebral bone mineral density, measures of muscle mass and diaphragm size, and vascular calcifications. These imaging biomarkers were consistently associated with more severe disease courses and worse clinical outcomes (49–56). Lower CT-derived vertebral BMD and imaging signs of sarcopenia were repeatedly linked to greater disease severity and poorer outcomes, while multiparametric models that combine these extrapulmonary features with clinical variables improved risk stratification beyond standard clinical assessment alone (51, 54, 55). Several reports also highlighted the feasibility of extracting multiple prognostic indicators from a single admission CT, suggesting a practical pathway to identify a frailty-like phenotype at presentation (52, 55). However, studies were predominantly retrospective and varied in methodology, patient selection, and measurement protocols, with age and comorbidity burden acknowledged as important potential confounders that complicate causal interpretation (50, 53). Taken together, the literature supports the potential clinical value of opportunistic CT-derived bone and muscle metrics for early prognostication in COVID-19, while underscoring the need for prospective validation and standardized imaging protocols before routine implementation.

Distinguishing glucocorticoid-induced osteoporosis from direct viral effects. Glucocorticoids and SARS-CoV-2 likely contribute to bone loss through partially distinct mechanisms. Glucocorticoid-induced osteoporosis is characterized predominantly by suppression of osteoblastogenesis and osteoblast survival, impaired osteocyte viability, reduced calcium absorption, and secondary alterations in PTH secretion, resulting in decreased bone formation and increased fracture risk (57, 58). In contrast, the direct skeletal effects of SARS-CoV-2 described in experimental models include productive or abortive infection of bone-remodeling cells, increased IL-6 and RANKL expression, and enhanced osteoclastogenesis (24, 25). The magnitude of post-COVID bone loss in individual patients therefore probably reflects the combined effects of viral inflammation, immobilization, nutritional deficiencies, and cumulative glucocorticoid exposure rather than a single pathogenic mechanism.

At the microstructural level, Buccino et al., using AI-assisted synchrotron imaging, found that larger, more spherical osteocyte lacunae increase microcrack formation and local stress, and similar microporosity changes in bones from prior COVID-19 patients suggest the infection may be associated with microstructural alterations that raise fragility (59).

Low BMD is a well-established predisposing factor for vertebral fractures, which represents a hallmark clinical manifestation of osteoporosis and impaired skeletal integrity. In line with this, numerous studies have reported a high prevalence of vertebral fractures among patients affected by COVID-19 (60–64). Additionally, vertebral BMD has been proposed as a predictor of COVID-19 outcomes (65, 66).

Although current evidence is insufficient to recommend routine skeletal screening for all individuals recovering from COVID-19, available clinical studies support a more proactive approach in selected high-risk populations (19, 20). Bone mineral density assessment by dual-energy X-ray absorptiometry (DXA) may be considered in patients with severe or prolonged COVID-19 requiring hospitalization, prolonged corticosteroid therapy, pre-existing osteopenia or osteoporosis, advanced age, prior fragility fractures, or persistent MSK symptoms after recovery (19, 20). Optimization of calcium and vitamin D status remains a fundamental component of bone health management, particularly in individuals with documented deficiency or increased fracture risk (19, 21). Patients receiving anti-osteoporotic therapy before SARS-CoV-2 infection should generally continue treatment whenever clinically appropriate, as current evidence does not support treatment discontinuation solely because of COVID-19. In addition, rehabilitation programs incorporating progressive weight-bearing and resistance exercises may help restore MSK function and counteract immobilization-associated bone loss (19, 21). Given the growing recognition that skeletal alterations may persist beyond the acute phase of infection, longitudinal clinical follow-up integrating fracture risk assessment, bone mineral density evaluation when indicated, and of bone turnover monitoring may facilitate early identification of patients who could benefit from targeted preventive or therapeutic interventions (19–21).

Long COVID is increasingly recognized as a condition that can extend well beyond the acute infection period and impact multiple aspects of health and quality of life. In a longitudinal cohort study, adults with long COVID exhibited persistent alterations in body composition—such as increased regional adiposity—and ongoing joint abnormalities over 12 months (43, 67).

### Conflicting clinical evidence

A recent longitudinal study found no evidence of accelerated BMD loss over 12 months in individuals with long COVID despite persistent MSK symptoms. Although these findings should be interpreted cautiously because of the relatively small sample size and heterogeneous study population, they suggest that SARS-CoV-2-associated bone loss may be predominantly an acute rather than a progressive process. Nevertheless, stable BMD does not exclude persistent alterations in bone remodeling or skeletal fragility, highlighting the need for longer-term studies evaluating bone quality, turnover markers, and fracture risk (68) (**Table 1**).

**Table 1:** Clinical and imaging evidence linking SARS-CoV-2 infection with alterations in bone metabolism and skeletal health.

Clinical finding	Clinical implications	Key limitations	Reference (s)
Elderly adults with insufficient VitD have a higher risk of SARS-CoV-2 infection; some COVID-19 patients later develop osteoporosis.	Heightened vigilance for bone health in people with osteoporosis; consider bone risk when planning COVID care.	Causality unclear (infection → osteoporosis vs shared risk factors).	(31)
Low vitamin D frequent in severe COVID-19; affects bone density, Ca;^2+^ homeostasis, immune response, and RAS (pathway used by virus).	Screen/treat vitamin D deficiency in at-risk patients; may influence both infection severity and bone outcomes.	Observational; reverse causality possible (sicker patients more deficient).	(32–35)
Acute hypocalcemia, low vitamin D with inadequate PTH response, increased vertebral fractures described in early reports.	Expect and monitor electrolyte and endocrine derangements (Ca, vit D, PTH) during acute illness.	Small/early studies; heterogeneity in measurement/timing.	(36)
Noticeable short-term reduction in BMD during/after hospitalization; proportion meeting osteoporosis criteria rose after hospitalization. Longer stays → greater loss; no major sex differences reported.	Hospitalized COVID-19 patients are a high-risk group for rapid bone loss—consider early assessment and follow-up.	Mostly observational, hospital populations (not generalizable to mild cases).	(39–43)
Hospitalized patients with musculoskeletal complaints showed higher prevalence of osteoporosis vs non-infected controls.	Targeted bone evaluation when hospitalized patients report MSK symptoms.	Selection bias (only symptomatic/hospitalized patients assessed).	(41)
Patients with osteopenia/osteoporosis on treatment still had further BMD reductions at lumbar spine and femur; more severe COVID → larger declines than milder disease.	Severe COVID survivors may need reassessment of osteoporosis therapy and monitoring of treatment efficacy.	Confounding by treatment (e.g., steroids) and comorbidities.	(39)
Months after clinical recovery, BMD remained lower than controls, implying effects can persist beyond acute illness.	Long-term bone follow-up is warranted in COVID survivors, not only during hospitalization.	Follow-up durations vary; baseline BMD often lacking.	(42)
Patients receiving corticosteroids during COVID care had greater lumbar/femoral BMD loss; effect related to cumulative dose and duration.	Minimize steroid exposure when possible; if used, implement bone-protective measures and monitoring.	Steroid indication/severity confounding (sicker → more steroids).	(40–42)
Admission CTs used opportunistically to measure vertebral BMD, muscle mass, diaphragm size, vascular calcifications; lower vertebral BMD and sarcopenia indicators associated with worse outcomes; multiparametric models improved prognostication.	Opportunistic CT metrics may help early risk stratification and identify a frailty-like phenotype at presentation.	Most studies retrospective, variable methodology; age/comorbidity confounding; need prospective validation and standardization.	(49–56)
AI-assisted synchrotron imaging found larger/more spherical osteocyte lacunae and microporosity changes in bones from prior COVID-19 patients that increase microcrack formation and fragility.	Suggests COVID-related microarchitectural damage could increase fragility beyond BMD loss.	Small, specialized imaging studies; mechanistic but limited clinical correlation so far.	(59)
Multiple studies report high vertebral fracture prevalence in COVID-affected patients; vertebral BMD proposed as a predictor of COVID outcomes.	Vertebral fractures both marker of skeletal fragility and potential prognostic indicator—consider opportunistic vertebral assessment.	Fracture ascertainment methods and timing vary; possible confounding by age/comorbidity.	(60–66)
Long COVID cohorts show persistent body composition changes (increased regional adiposity) and joint abnormalities up to 12 months; no clear evidence of accelerated BMD loss vs well-recovered controls in one longitudinal cohort.	Continue musculoskeletal monitoring (including sarcopenia and joint health); BMD loss may not accelerate in all long COVID patients but individualized follow-up needed.	Limited long-term data; heterogeneous long COVID phenotypes.	(43, 67, 68)

Summary of clinical, biochemical, imaging, and microstructural findings reporting changes in bone mineral density, vertebral fracture prevalence, osteometabolic disturbances, and CT-derived musculoskeletal biomarkers in patients with acute COVID-19, post-acute recovery, and long COVID. Associations with disease severity, hospitalization, corticosteroid exposure, and imaging-based prognostic indicators are highlighted. BMD, Bone Mineral Density; MSK, Musculoskeletal; RAS, Renin-Angiotensin System; PTH, Parathyroid Hormone; VitD, Vitamin D.

Although the overall clinical evidence suggests that SARS-CoV-2 infection may adversely affect skeletal health, the findings remain heterogeneous across studies (39, 40, 43, 66). Studies evaluating bone mineral density and fracture risk have reported reductions in BMD and an increased incidence of osteoporotic fractures following COVID-19, particularly among patients with severe disease or prolonged follow-up (39, 40, 43, 60–66). In contrast, several opportunistic chest CT studies primarily demonstrate that pre-existing low vertebral BMD predicts COVID-19 severity and mortality, rather than establishing that SARS-CoV-2 directly causes bone loss (49–56, 65). Furthermore, Kerschan-Schindl et al. observed reduced bone turnover markers in patients recovering from moderate COVID-19, suggesting that skeletal responses may vary according to disease stage and timing of assessment (3). Likewise, Alghamdi et al. found no accelerated BMD loss during 12 months of follow-up in individuals with long COVID, although the relatively small sample size and heterogeneous patient population may have limited the detection of subtle skeletal changes (68). Importantly, these apparently divergent findings are not necessarily contradictory but likely reflect differences in study design, patient characteristics, disease severity, follow-up duration, and the skeletal outcomes evaluated, underscoring the need for larger prospective longitudinal studies with standardized assessment protocols (3, 20, 68).

## Proposed practical framework for post-COVID bone health monitoring

Although current evidence does not support universal skeletal screening after SARS-CoV-2 infection, available clinical data suggest that an individualized risk-based approach may facilitate early identification of patients who are most likely to benefit from bone health assessment. Based on the clinical and mechanistic evidence reviewed herein, we propose the following pragmatic framework for post-COVID skeletal monitoring.

Patients should first be stratified according to their risk profile. Individuals with severe COVID-19 requiring hospitalization, prolonged systemic corticosteroid treatment, advanced age, pre-existing osteopenia or osteoporosis, previous fragility fractures, vitamin D deficiency, prolonged immobilization, or persistent MSK symptoms after recovery should be considered at increased risk for adverse skeletal outcomes.

For low-risk individuals who experienced mild disease without major risk factors, reinforcement of general bone health measures—including adequate calcium and vitamin D intake, regular weight-bearing exercise, smoking cessation, and periodic clinical reassessment if symptoms develop—appears appropriate.

In contrast, patients classified as high risk should undergo a comprehensive skeletal evaluation approximately 3–6 months after recovery. This assessment may include fracture risk estimation (e.g., FRAX where appropriate), dual-energy X-ray absorptiometry (DXA), laboratory evaluation of calcium, phosphate, 25-hydroxyvitamin D, parathyroid hormone, and bone turnover markers when clinically indicated. Opportunistic analysis of vertebral bone mineral density from chest CT scans obtained during acute COVID-19 may provide complementary information when such imaging is already available.

Patients with confirmed osteoporosis, significant bone loss, or high fracture risk should receive management according to current osteoporosis guidelines. This should include optimization of calcium and vitamin D status, continuation or initiation of anti-osteoporotic therapy when indicated, and structured rehabilitation with progressive weight-bearing and resistance exercise. Patients with normal bone mineral density but persistent risk factors may benefit from repeat clinical evaluation and reassessment of bone mineral density after 12–24 months depending on their overall risk profile.

**Figure 1:**
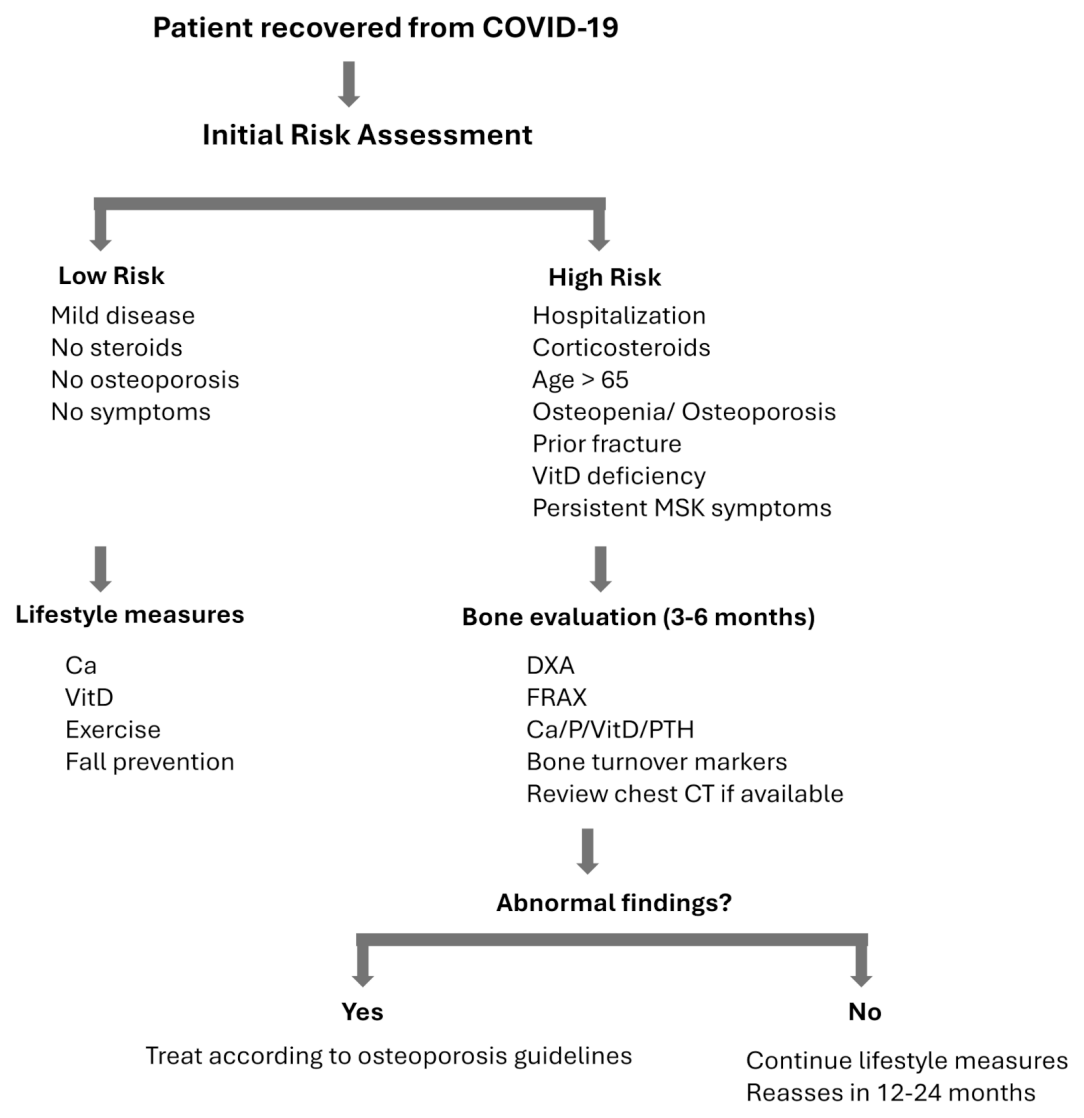
Proposed algorithm for bone health assessment and management following recovery from COVID-19. Patients recovering from COVID-19 should undergo an initial risk assessment based on disease severity, corticosteroid exposure, age, baseline bone health, fracture history, vitamin D (VitD) status, and the presence of persistent musculoskeletal symptoms. Individuals at low risk should receive lifestyle measures, including adequate calcium and vitD intake, regular exercise, and fall prevention strategies. Patients at high risk should undergo bone health evaluation within 3–6 months, including dual-energy X-ray absorptiometry (DXA), fracture risk assessment (FRAX), laboratory evaluation (serum calcium (Ca), phosphate (P), vitD, and parathyroid hormone (PTH)), measurement of bone turnover markers, and review of chest computed tomography (CT), if available, for opportunistic assessment of vertebral fractures or bone density. Patients with abnormal findings should be treated according to established osteoporosis guidelines, whereas those without abnormalities should continue lifestyle measures and undergo reassessment after 12–24 months.

This proposed framework is intended to translate the currently available evidence into a practical clinical approach. Because existing data are predominantly observational, prospective studies are required to validate the optimal timing, frequency, and cost-effectiveness of post-COVID skeletal surveillance (**Figure 1**).

## Bone dynamics in homeostasis

Bone homeostasis is an active, tightly regulated process that preserves skeletal strength and mineral balance by coordinating bone formation and resorption. Osteoblasts are derived from MSC and are responsible for synthesizing and mineralizing the organic bone matrix. Their differentiation and function are regulated by lineage-defining transcription factors, including RUNX2 and Osterix (69). Their proliferation, survival and maturation are promoted by anabolic signaling cascades, including Wnt/β-catenin, BMP/TGF-β, PI3K/AKT and IGF pathways, which together drive production of matrix proteins (type I collagen, osteocalcin) and the enzyme ALP required for mineral deposition (70–73). Opposing this anabolic program, osteoclasts arise from hematopoietic monocyte–macrophage precursors and, after fusion into multinucleated cells, resorb mineralized bone by acidification of the resorption lacuna and secretion of proteolytic enzymes such as cathepsin K and matrix metalloproteinases (74–76). Osteoclastogenesis is critically dependent on M-CSF and RANKL signaling (77, 78).

Embedded within the mineralized matrix, osteocytes originate when osteoblasts become entrapped and mature into a long-lived, dendritic cell population that senses and integrates mechanical and biochemical cues (79). Through their lacuno–canalicular network, they detect loading and microenvironmental changes, transducing these signals into paracrine factors that modulate both bone formation and resorption (79). Osteocytes are a principal source of RANKL and its decoy receptor OPG, thereby influencing osteoclastogenesis, and they secrete Wnt inhibitors such as sclerostin and DKK1 to restrain osteoblastic activity (80, 81). Collectively, these outputs allow osteocytes to couple mechanical and metabolic information to finely tune bone turnover.

At the molecular level, the RANKL–RANK–OPG axis constitutes the core regulatory module controlling resorption: RANKL produced by stromal cells, osteoblasts, osteocytes and immune cells engages receptor activator of nuclear factor κB (RANK) on osteoclast precursors to activate downstream effectors such as nuclear factor kappa-light-chain-enhancer of activated B cells (NF-κB), mitogen-activated protein kinase (MAPK) and nuclear factor of activated T cells, Cytoplasmic 1 (NFATc1) that drive differentiation and resorptive function, while OPG restrains this process by sequestering RANKL (82–84). This axis is modulated by additional receptors (e.g., LGR4), systemic hormones (parathyroid hormone, vitamin D, estrogen, glucocorticoids) and immune-derived cytokines, reflecting the close integration of skeletal remodeling with endocrine and immune systems (85, 86).

The dialogue between the immune system and the skeleton underlies the bone system’s ability to adapt. The term *osteoimmunology* was originally introduced by Arron and Choi and was subsequently established as a distinct interdisciplinary field through the seminal work of Takayanagi and colleagues (87, 88). This interdisciplinary field has, over the past two decades, revealed the central role of the RANKL–OPG pathway in maintaining skeletal balance—RANKL being a principal driver of osteoclast formation. Besides osteoblasts, immune cells such as activated T and B lymphocytes also express RANKL and contribute to the development of bone disorders including osteoporosis. Experimental studies demonstrate that proinflammatory cytokines, including IL-1β, IL-6, IL-17, and TNF-α, drive bone resorption by upregulating RANK–RANKL signaling, thereby contributing to various bone disorders (89, 90). Consequently, when these signaling networks are disrupted by aging, hormonal imbalances, chronic inflammation, or infectious insults, the normal coupling of bone formation and resorption is lost, leading to net bone loss as seen in osteoporosis, inflammatory osteolysis, and infection-associated bone disease (91, 92). Accordingly, recent findings in COVID-19 patients with severe disease, exhibit elevated proinflammatory cytokines and an increased RANKL/OPG ratio, reflecting a shift toward enhanced osteoclast activity (93).

Clarifying these mechanisms has informed current targeted therapies and provides a mechanistic framework to assess how emerging pathogens, including SARS-CoV-2, might undermine skeletal integrity.

## Direct and indirect effects of SARS-CoV-2 on bone remodeling cells

Beyond serving as a viral entry receptor, ACE2 is a functional component of the local RAS within bone. ACE2 converts angiotensin II to angiotensin-(1–7), thereby favoring signaling through the Mas receptor, which has been associated with enhanced osteoblast activity and attenuation of osteoclastogenesis (22, 42). Experimental studies indicate that excess angiotensin II promotes oxidative stress, inflammation, and bone resorption, whereas activation of the ACE2/Ang-(1–7)/Mas axis exerts protective skeletal effects (22, 94, 95). Consequently, SARS-CoV-2-mediated downregulation or dysregulation of ACE2 could theoretically shift the local bone environment toward a pro-resorptive state independent of direct cellular infection, providing an additional endocrine–skeletal mechanism linking COVID-19 and bone loss (101–103).

SARS-CoV-2 has been implicated in skeletal abnormalities through both direct and indirect mechanisms affecting bone remodeling. Viral entry is primarily mediated by the ACE2 receptor, which is widely expressed in multiple tissues, including bone cells involved in bone turnover. Early evidence suggested that SARS-CoV-2 could influence bone metabolism through disruption of the ACE2/Ang-(1-7)/Mas receptor axis, a key regulator of osteoblast and osteoclast activity. Supporting this hypothesis, studies during the SARS-CoV outbreak identified ACE2 expression in CD14^+^ monocytes, the precursors of osteoclasts, indicating that cells of the osteoclast lineage may be susceptible to coronavirus infection (7). Furthermore, the SARS-CoV accessory protein 3a/X1 was shown to promote osteoclast differentiation by inducing tumor necrosis factor-α (TNF-α) in human cell lines and receptor activator of nuclear factor κB ligand (RANKL) in murine cell lines, providing early evidence that coronaviruses can directly modulate bone remodeling (7).

In addition, neuropilin-1 (NRP1) has recently been identified as an alternative entry receptor in bone marrow-derived macrophages from mice, suggesting that viral tropism may differ among bone cell populations (23). Productive infection of differentiated human osteoblasts and abortive infection of MSC and osteoclast precursors alter osteoblast differentiation, increase IL-6 and RANKL expression, enhance osteoclastogenesis, and ultimately shift bone remodeling toward bone resorption (24, 25). These direct virus-specific effects are further amplified by viral proteins, including Spike and ORF8, and by virus-induced microRNAs such as miR-4485-3p, which promote inflammatory and pro-osteoclastogenic signaling (24, 26–28).

Subsequent studies extended these observations to SARS-CoV-2. Recombinant Spike protein has been shown to induce a senescence-associated secretory phenotype (SASP) in murine bone marrow-derived macrophages, increasing the expression of pro-inflammatory cytokines and cathepsins in M-CSF and IL-34-differentiated macrophages (26). Additionally, the detection of SARS-CoV-2 viral RNA in post-mortem bone marrow samples provides evidence that the virus can disseminate to the bone marrow, supporting the biological plausibility of direct interactions with cells of the bone microenvironment (29).

**Figure 2:**
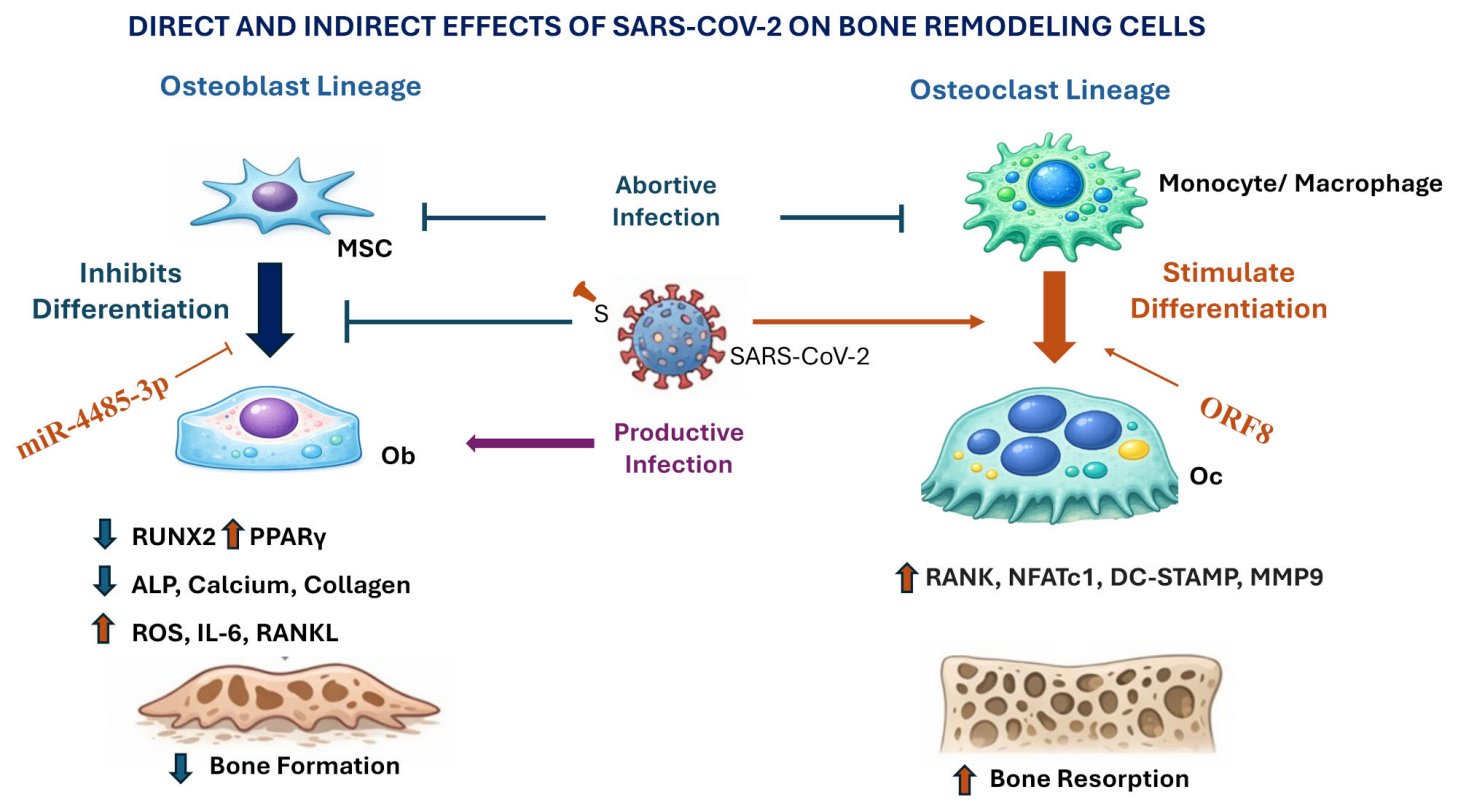
Direct and indirect effects of SARS-CoV-2 on bone-remodeling cells. Schematic summarizing proposed pathways by which SARS-CoV-2 may alter skeletal homeostasis through actions on cells of the osteoblast and osteoclast lineages. Solid arrows indicate stimulatory influences and flat-headed lines indicate inhibitory influences. Direct viral effects are represented as productive or abortive infection of susceptible cells; viral proteins (S, ORF8) and virus-induced microRNA (miR-4485-3p) are shown as modulators of cell fate and function. On the osteoblast lineage (left), SARS-CoV-2–associated signals are linked to reduced osteoblastic differentiation and function (decreased RUNX2, ALP, collagen deposition, and calcium handling) and to up-regulation of stress/inflammatory mediators (ROS, IL-6) that impair bone formation. On the osteoclast lineage (right), abortive infection of monocyte/macrophage precursors directly promotes osteoclast differentiation via increased RANK, NFATc1, DC-STAMP and MMP9 expression, even in the absence of RANKL, while inflammatory signals further enhance osteoclastogenesis and bone resorption.

Osteoblasts and their mesenchymal progenitors are central regulators of bone remodeling, controlling bone formation and osteoclast differentiation primarily through the RANKL/OPG axis, and perturbation of osteoblast function or progenitor fate can shift remodeling toward pathological resorption (**Figure 2**). Emerging evidence indicates that SARS-CoV-2 alters both osteoblast-lineage cells and osteoclast precursors by multiple, complementary mechanisms. our previously published findings *In vitro* studies from our previously published findings show that ancestral (Wuhan) and Omicron BA.5 SARS-CoV-2 productively infect differentiated human osteoblasts while producing an abortive infection in MSCprecursors; although cell viability is preserved, viral exposure during osteogenic differentiation markedly impairs osteoblast function (reduced ALP activity, calcium and collagen deposition) and increases mitochondrial reactive oxygen species (ROS). At the transcriptional level, infection causes early RUNX2 downregulation with later PPARγ upregulation (increasing the PPARγ/RUNX2 ratio), stimulates IL-6 secretion, and increases RANKL expression, changes that collectively suppress osteogenesis and favor a pro-osteoclastogenic milieu. That UV-inactivated virus reproduces these inhibitory effects, and that neutralizing anti-Spike antibodies attenuate them, implicating a structural role for Spike in mediating osteoblast dysfunction (24).

Hsa-miR-4485-3p is a 20-nucleotide mature miRNA processed from the hsa-mir-4485 precursor. It has been associated with cancer (96), infectious diseases (97, 98), and bone-fracture healing in COVID-19 patients (27). hsa-miR-4485-3p has been shown to inhibit osteogenesis and to promote adipogenesis. Recovered (IgG-positive) individuals show marked upregulation of hsa-miR-4485-3p, which impairs osteogenic differentiation of human bone marrow stromal cells *in vitro* by targeting TLR4. In a murine femoral fracture model, overexpression of hsa-miR-4485-3p delayed callus formation, whereas its inhibition rescued osteogenesis and improved repair (27).

Viral proteins can also directly drive osteoclastogenic signaling. The accessory protein ORF8 is associated with a proinflammatory, pro-osteoclastogenic plasma profile, characterized by elevated bone-resorption markers and an increased RANKL/OPG ratio, in patients with immune-mediated inflammatory diseases. *In vitro*, ORF8 stimulates osteoblasts from patients with rheumatoid arthritis to produce IL-6, TNF, and IL-17 family cytokines and to upregulate RANKL. Conditioned media from ORF8-treated osteoblasts markedly enhances osteoclast differentiation from myeloid precursors (28).

SARS-CoV-2 also acts directly on the osteoclast lineage. We have demonstrated that in human monocyte-derived macrophages (osteoclast precursors) the virus establishes an abortive infection (transient viral RNA, ACE2 upregulation, no productive virion release), yet viral exposure accelerates osteoclastogenesis in a dose-dependent manner, increasing tartrate-resistant acid phosphatase (TRAP)-positive multinucleated cells, maturation markers (CD51/61), resorptive activity and expression of osteoclast genes (RANK, NFATc1, dendritic cell-specific transmembrane protein (DC-STAMP), matrix metalloproteinase-9 (MMP9). Mechanistically, an early M1-like cytokine response (TNF-α, IL-1β, IL-6) later shifts toward an M2/TGF-β/IL-10 milieu that permits RANKL-independent osteoclast formation, with TGF-β facilitating TNF-driven osteoclastogenesis (25) (**Figure 2**).

Complementing these human data, murine studies demonstrate species-specific modes of myeloid infection. Using an Ad5-hACE2 model, Gao et al. reported that SARS-CoV-2 productively infects murine bone marrow-derived macrophages (BMMs) via neuropilin-1 (NRP1) rather than ACE2. NRP1-high (Cx3cr1^+^Ccr2^+^) macrophage subsets were preferentially infected, whereas NRP1 expression and infectivity declined during osteoclast differentiation. Moreover, SARS-CoV-2 infection impaired RANKL-driven osteoclastogenesis both *ex vivo* and *in vivo* (23, 99).

These cellular perturbations are reflected in multiple animal models: intranasal SARS-CoV-2 infection of transgenic hACE2 and K18-hACE2 mice produces rapid, osteoclast-driven trabecular bone loss (reduced trabecular mass, number and thickness; increased separation and osteoclast surface), sometimes with detectable viral proteins or hACE2 in bone and joint tissues, while golden Syrian hamsters show rapid, systemic and persistent trabecular loss driven primarily by inflammation-induced osteoclastogenesis even in the absence of detectable viral RNA in skeletal compartments (100–102), (**Figure 2**).

Taken together, these findings are not necessarily contradictory but instead highlight species-, receptor-, and differentiation stage-specific mechanisms of SARS-CoV-2 infection in the osteoclast lineage. Human osteoclast precursors appear to rely predominantly on ACE2-mediated viral interactions, in which abortive infection promotes inflammatory cytokine production and RANKL-independent osteoclastogenesis. In contrast, Gao et al. demonstrated that murine bone marrow-derived macrophages are infected primarily through NRP1, with preferential infection of NRP1-high macrophage subsets and progressive loss of NRP1 expression during osteoclast differentiation, thereby limiting infection of mature osteoclasts and impairing RANKL-driven osteoclastogenesis (23). ACE2 binds the Receptor Binding Domain (RBD) and NRP1 binds the C-end Rule (CendR) motif exposed after the Spike protein is cleaved by furin in human cells. NRP1 is a potent attachment/uptake cofactor (helping with the spread of the virus into cells), but studies indicate that in primary human macrophages, ACE2 is the essential anchor that initiates the viral fusion process and triggers innate immune sensing. In murine macrophages, CendR-NRP1 interaction alone is sufficient to induce internalization, even in the absence of ACE2, but this results in abortive replication due to downstream host restrictions (103). These divergent outcomes likely reflect differences in receptor usage, species-specific receptor expression patterns, the stage of myeloid differentiation at which infection occurs, and the experimental models employed, rather than fundamentally opposing mechanisms. Importantly, although the direct effects on osteoclast differentiation differ between the human and murine systems, both models ultimately support the concept that SARS-CoV-2 perturbs bone remodeling. Consequently, findings from murine models should be interpreted cautiously when extrapolating mechanisms of viral tropism and osteoclast regulation to human skeletal biology.

Together, these data suggest that SARS-CoV-2 may perturb bone homeostasis through direct effects on osteoblasts and progenitors, induction of pro-resorptive microRNAs and accessory proteins, and promotion of osteoclastogenesis via inflammatory cytokine milieus — supporting the concept that infection-associated inflammatory bone loss is an underrecognized extrapulmonary consequence of COVID-19 and underscoring the need for longer-term skeletal monitoring and targeted interventions.

## Lessons from HIV-associated bone disease

Although SARS-CoV-2 and HIV differ substantially in their clinical course and viral persistence, both infections share mechanisms that may contribute to skeletal deterioration, including chronic immune activation, increased production of pro-inflammatory cytokines, and dysregulation of the RANKL/OPG axis. In HIV infection, these pathways, together with antiretroviral therapy, are well-recognized contributors to osteoporosis and fracture risk (104–108). In contrast, current evidence suggests that SARS-CoV-2-associated bone loss is driven primarily by acute inflammation, direct effects on bone-remodeling cells, immobilization, and corticosteroid exposure (4, 5, 18, 31). Lessons from HIV research highlight the importance of longitudinal skeletal monitoring, evaluation of bone turnover biomarkers, and early identification of high-risk individuals, approaches that may also prove valuable in patients recovering from COVID-19.

## Limitations and future directions

While preclinical models provide mechanistic insights, species-specific viral tropism complicates translation: SARS-CoV-2 enters human osteoblasts and monocyte precursors primarily via ACE2, promoting osteoclastogenesis, whereas murine myeloid cells rely on NRP1, often yielding inhibitory effects on differentiation. Human data are further confounded by corticosteroids, vitamin D deficiency, age/comorbidities (unadjusted in most CT studies), and retrospective designs lacking pre-infection baselines, precluding definitive causality. Prospective cohorts with standardized imaging, biomarker stratification (e.g., RANKL/OPG ratios), and humanized models are urgently needed to disentangle direct viral from indirect inflammatory effects and guide interventional trials (4, 40).

## Concluding remarks

Accumulating clinical, cellular and animal evidence suggests that SARS-CoV-2 infection may perturb skeletal homeostasis by converging mechanisms that both suppress bone formation and accelerate bone resorption. Clinical cohorts and opportunistic imaging studies report associations between acute and persistent reductions in bone mineral density, an increased prevalence of vertebral fractures and CT-derived MSK signatures that predict worse outcomes. Mechanistic work *in vitro* and *in vivo* implicates direct viral effects on osteoblasts and osteoprogenitors, virus- and host-derived mediators (e.g., ORF8, pro-resorptive microRNAs) and dysregulated cytokine milieus that together increase the RANKL/OPG ratio and promote osteoclastogenesis. Species-specific differences in viral tropism of myeloid cells (ACE2 vs NRP1) and divergent outcomes in experimental models caution against simple extrapolation but nonetheless support a consistent pathophysiologic theme: COVID-19 may contribute to inflammatory bone loss that may persist beyond the acute illness.

These findings have immediate clinical and research implications. Clinicians should maintain a high index of suspicion for adverse skeletal outcomes after COVID-19, particularly in patients with severe disease, prolonged corticosteroid exposure, or pre-existing low BMD. Early bone health assessment and fracture prevention strategies should be considered in these high-risk individuals, where appropriate. From a translational perspective, the mechanistic links described here identify candidate biomarkers (RANKL/OPG, specific microRNAs, circulating viral antigens) and therapeutic targets (TNF/RANKL pathways, neutralizing anti-Spike strategies) that warrant prospective evaluation.

Beyond improving our understanding of disease pathogenesis, the available evidence has important implications for clinical practice. Although definitive evidence-based recommendations are still evolving, clinicians should remain vigilant for adverse skeletal consequences following COVID-19, particularly among older adults and individuals with severe disease, prolonged immobilization, corticosteroid exposure, vitamin D deficiency, or pre-existing osteoporosis. In these populations, individualized fracture risk assessment and consideration of bone mineral density evaluation may facilitate early identification of patients at increased risk of skeletal deterioration. Optimization of calcium and vitamin D status, maintenance of appropriate osteoporosis therapy, promotion of physical rehabilitation and weight-bearing exercise, and adherence to current osteoporosis management guidelines represent practical measures that may help preserve skeletal health during recovery.

Beyond optimization of calcium and vitamin D status, the molecular mechanisms described in this review suggest that several existing osteoporosis therapies may warrant evaluation in patients at high risk of post-COVID skeletal deterioration. The consistent increase in the RANKL/OPG ratio, enhanced osteoclastogenesis, and elevated inflammatory cytokine production provide a strong biological rationale for investigating anti-resorptive therapies, particularly the anti-RANKL monoclonal antibody denosumab, in appropriately selected patients. Likewise, bisphosphonates may help limit the accelerated osteoclast-mediated bone resorption observed following SARS-CoV-2 infection. Although no clinical evidence currently supports routine use of these agents specifically to prevent COVID-19-associated bone loss, patients who already meet established indications for osteoporosis treatment should receive standard guideline-based therapy. Furthermore, the suppression of osteoblast differentiation induced by SARS-CoV-2 raises the possibility that anabolic agents, including teriparatide, abaloparatide, or romosozumab, could have therapeutic value in patients with persistent impairment of bone formation, although this remains entirely speculative. Finally, because inflammatory cytokines (TNF-α, IL-1β, IL-6, and IL-17) contribute to the pro-osteoclastogenic environment, future studies should determine whether immunomodulatory strategies capable of attenuating these pathways may also provide skeletal benefits in selected post-COVID populations.

Future prospective longitudinal studies are needed to establish the incidence, duration, and clinical significance of post-COVID bone loss, identify reliable biomarkers of skeletal involvement, and determine whether specific anti-resorptive or anabolic therapies can prevent or reverse SARS-CoV-2-associated bone deterioration. Standardized imaging protocols, integration of bone turnover biomarkers, and randomized clinical trials evaluating targeted interventions will be essential to define evidence-based strategies for post-COVID skeletal surveillance and management. As our understanding of the interaction between viral infection, inflammation, and bone remodeling continues to evolve, incorporation of bone health assessment into the long-term care of selected COVID-19 survivors may contribute to reducing fracture risk and improving overall functional outcomes.

Pending prospective validation, a risk-stratified monitoring strategy such as the framework proposed in this review may facilitate early identification of patients at increased risk of post-COVID skeletal deterioration while avoiding unnecessary screening of low-risk individuals.

## References

[1] Zheng KI Feng G Liu WY Targher G Byrne CD Zheng MH . Extrapulmonary complications of covid-19: a multisystem disease? J Med Virol. (2021) 93:323–35. doi: 10.1002/jmv.26294 32648973 PMC7405144

[2] Schultze JL Aschenbrenner AC . Covid-19 and the human innate immune system. Cell. (2021) 184:1671–92. doi: 10.1016/j.cell.2021.02.029 33743212 PMC7885626

[3] Kerschan-Schindl K Dovjak P Butylina M Rainer A Mayr B Roggla V . Moderate covid-19 disease is associated with reduced bone turnover. J Bone Miner Res. (2023) 38:943–50. doi: 10.1002/jbmr.4822 37126438

[4] Creecy A Awosanya OD Harris A Qiao X Ozanne M Toepp AJ . Covid-19 and bone loss: a review of risk factors, mechanisms, and future directions. Curr Osteoporos Rep. (2024) 22:122–34. doi: 10.1007/s11914-023-00842-2 38221578 PMC10912142

[5] Harris A Creecy A Awosanya OD McCune T Ozanne MV Toepp AJ . Sars-cov-2 and its multifaceted impact on bone health: mechanisms and clinical evidence. Curr Osteoporos Rep. (2024) 22:135–45. doi: 10.1007/s11914-023-00843-1 38236510 PMC10912131

[6] Shao X Zhang X Zhang R Zhu R Hou X Yi W . The atlas of ace2 expression in fetal and adult human hearts reveals the potential mechanism of heart-injured patients infected with sars-cov-2. Am J Physiol Cell Physiol. (2022) 322:C723–38. doi: 10.1152/ajpcell.00169.2021 35138176 PMC8977135

[7] Obitsu S Ahmed N Nishitsuji H Hasegawa A Nakahama K Morita I . Potential enhancement of osteoclastogenesis by severe acute respiratory syndrome coronavirus 3a/x1 protein. Arch Virol. (2009) 154:1457–64. doi: 10.1007/s00705-009-0472-z 19685004 PMC7086770

[8] Devaux CA Pinault L Osman IO Raoult D . Can ace2 receptor polymorphism predict species susceptibility to sars-cov-2? Front Public Health. (2020) 8:608765. doi: 10.3389/fpubh.2020.608765 33643982 PMC7902720

[9] Devaux CA Rolain JM Raoult D . Ace2 receptor polymorphism: susceptibility to sars-cov-2, hypertension, multi-organ failure, and covid-19 disease outcome. J Microbiol Immunol Infect. (2020) 53:425–35. doi: 10.1016/j.jmii.2020.04.015 32414646 PMC7201239

[10] Maffia-Bizzozero S Cevallos C Lenicov FR Freiberger RN Lopez CAM Guano Toaquiza A . Viable sars-cov-2 omicron sub-variants isolated from autopsy tissues. Front Microbiol. (2023) 14:1192832. doi: 10.3389/fmicb.2023.1192832 37283920 PMC10240073

[11] Deinhardt-Emmer S Wittschieber D Sanft J Kleemann S Elschner S Haupt KF . Early postmortem mapping of sars-cov-2 rna in patients with covid-19 and the correlation with tissue damage. Elife. (2021) 10:1–22. doi: 10.7554/eLife.60361 33781385 PMC8009677

[12] Zuo W He D Liang C Du S Hua Z Nie Q . The persistence of sars-cov-2 in tissues and its association with long covid symptoms: a cross-sectional cohort study in China. Lancet Infect Dis. (2024) 24:845–55. doi: 10.1016/S1473-3099(24)00171-3 38663423

[13] Goh D Lim JCT Fernaindez SB Joseph CR Edwards SG Neo ZW . Case report: persistence of residual antigen and rna of the sars-cov-2 virus in tissues of two patients with long covid. Front Immunol. (2022) 13:939989. doi: 10.3389/fimmu.2022.939989 36131932 PMC9483160

[14] El-Baky NA Amara AA Uversky VN Redwan EM . Intrinsic factors behind long covid: iii. persistence of sars-cov-2 and its components. J Cell Biochem. (2024) 125:22–44. doi: 10.1002/jcb.30514 38098317

[15] de La Porte des Vaux C Veyrenche N Silva KD Chavarot N Burgard M Paccoud O . Immunocompromised patients with persistent sars-cov-2 viral shedding &gt;/=8 weeks, clinical outcomes, and virological dynamics: a retrospective multicenter cohort study, 2020-2024. Antimicrob Agents Chemother. (2025) 69:e0065825. doi: 10.1128/aac.00658-25 41004259 PMC12587602

[16] Swank Z Karlson EW Walt DR . 'Measurement of circulating viral antigens post-sars-cov-2 infection in a multicohort study' - author's reply. Clin Microbiol Infect. (2025) 31:484–5. doi: 10.1016/j.cmi.2024.10.028 39489294

[17] Manrique JM Maffia-Bizzozero S Delpino MV Quarleri J Jones LR . Multi-organ spread and intra-host diversity of sars-cov-2 support viral persistence, adaptation, and a mechanism that increases evolvability. J Med Virol. (2024) 96:e70107. doi: 10.1002/jmv.70107 39654307 PMC11656291

[18] Galliera E Massaccesi L Mangiavini L De Vecchi E Villa F Corsi Romanelli MM . Effects of covid-19 on bone fragility: a new perspective from osteoimmunological biomarkers. Front Immunol. (2024) 15:1493643. doi: 10.3389/fimmu.2024.1493643 39582872 PMC11582977

[19] Hu CL Zheng MJ He XX Liu DC Jin ZQ Xu WH . Covid-19 and bone health. Eur Rev Med Pharmacol Sci. (2023) 27:3191–200. doi: 10.26355/eurrev_202304_31953 37070922

[20] Alghamdi F Mokbel K Meertens R Obotiba AD Alharbi M Knapp KM . Bone mineral density, bone biomarkers, and joints in acute, post, and long covid-19: a systematic review. Viruses. (2024) 16:1–21. doi: 10.3390/v16111694 39599809 PMC11599111

[21] Foessl I Dimai HP Obermayer-Pietsch B . Long-term and sequential treatment for osteoporosis. Nat Rev Endocrinol. (2023) 19:520–33. doi: 10.1038/s41574-023-00866-9 37464088

[22] Queiroz-Junior CM Santos A Galvao I Souto GR Mesquita RA Sa MA . The angiotensin converting enzyme 2/angiotensin-(1-7)/mas receptor axis as a key player in alveolar bone remodeling. Bone. (2019) 128:115041. doi: 10.1016/j.bone.2019.115041 31442676

[23] Gao J Mei H Sun J Li H Huang Y Tang Y . Neuropilin-1-mediated sars-cov-2 infection in bone marrow-derived macrophages inhibits osteoclast differentiation. Adv Biol (Weinh). (2022) 6:e2200007. doi: 10.1002/adbi.202200007 35195371 PMC9073998

[24] Freiberger RN Lopez CAM Jarmoluk P Palma MB Cevallos C Sviercz FA . Sars-cov-2 impairs osteoblast differentiation through spike glycoprotein and cytokine dysregulation. Viruses. (2025) 17:1–20. doi: 10.3390/v17020143 40006897 PMC11860324

[25] Sviercz F Jarmoluk P Godoy Coto J Cevallos C Freiberger RN Lopez CAM . The abortive sars-cov-2 infection of osteoclast precursors promotes their differentiation into osteoclasts. J Med Virol. (2024) 96:e29597. doi: 10.1002/jmv.29597 38587211

[26] Duarte C Akkaoui J Ho A Garcia C Yamada C Movila A . Age-dependent effects of the recombinant spike protein/sars-cov-2 on the m-csf- and il-34-differentiated macrophages *in vitro*. Biochem Biophys Res Commun. (2021) 546:97–102. doi: 10.1016/j.bbrc.2021.01.104 33578295 PMC7857081

[27] Mi B Xiong Y Zhang C Zhou W Chen L Cao F . Sars-cov-2-induced overexpression of mir-4485 suppresses osteogenic differentiation and impairs fracture healing. Int J Biol Sci. (2021) 17:1277–88. doi: 10.7150/ijbs.56657 33867845 PMC8040480

[28] Melano I Azamor T Caetano CC Meyer NM Onwubueke C Visperas A . Sars-cov-2 orf8 drives osteoclastogenesis in preexisting immune-mediated inflammatory diseases. JCI Insight. (2024) 9:1–17. doi: 10.1172/jci.insight.178820 39704167 PMC11665583

[29] Jurek T Rorat M Szleszkowski L Tokarski M Pielka I Malodobra-Mazur M . Sars-cov-2 viral rna is detected in the bone marrow in post-mortem samples using rt-lamp. Diagn (Basel). (2022) 12:1–5. doi: 10.3390/diagnostics12020515 35204605 PMC8870857

[30] Sozen T Ozisik L Basaran NC . An overview and management of osteoporosis. Eur J Rheumatol. (2017) 4:46–56. doi: 10.5152/eurjrheum.2016.048 28293453 PMC5335887

[31] Liu F Song C Cai W Chen J Cheng K Guo D . Shared mechanisms and crosstalk of covid-19 and osteoporosis via vitamin d. Sci Rep. (2022) 12:18147. doi: 10.1038/s41598-022-23143-7 36307516 PMC9614744

[32] Charoenngam N Holick MF . Immunologic effects of vitamin d on human health and disease. Nutrients. (2020) 12:1–28. doi: 10.3390/nu12072097 32679784 PMC7400911

[33] Hurst EA Mellanby RJ Handel I Griffith DM Rossi AG Walsh TS . Vitamin d insufficiency in covid-19 and influenza a, and critical illness survivors: a cross-sectional study. BMJ Open. (2021) 11:e055435. doi: 10.1136/bmjopen-2021-055435 34686560 PMC8728359

[34] Parra-Ortega I Alcara-Ramirez DG Ronzon-Ronzon AA Elias-Garcia F Mata-Chapol JA Cervantes-Cote AD . 25-hydroxyvitamin d level is associated with mortality in patients with critical covid-19: a prospective observational study in Mexico city. Nutr Res Pract. (2021) 15:S32–40. doi: 10.4162/nrp.2021.15.S1.S32 34909131 PMC8636388

[35] Kumar R Rathi H Haq A Wimalawansa SJ Sharma A . Putative roles of vitamin d in modulating immune response and immunopathology associated with covid-19. Virus Res. (2021) 292:198235. doi: 10.1016/j.virusres.2020.198235 33232783 PMC7680047

[36] di Filippo L Frara S Giustina A . The emerging osteo-metabolic phenotype of covid-19: clinical and pathophysiological aspects. Nat Rev Endocrinol. (2021) 17:445–6. doi: 10.1038/s41574-021-00516-y 34079100 PMC8170860

[37] Martineau AR Forouhi NG . Vitamin d for covid-19: a case to answer? Lancet Diabetes Endocrinol. (2020) 8:735–6. doi: 10.1016/S2213-8587(20)30268-0 32758429 PMC7398646

[38] Silva MC Furlanetto TW . Does serum 25-hydroxyvitamin d decrease during acute-phase response? a systematic review. Nutr Res. (2015) 35:91–6. doi: 10.1016/j.nutres.2014.12.008 25631715

[39] Elmedany SH Badr OI Abu-Zaid MHT Tabra SAA . Bone mineral density changes in osteoporotic and osteopenic patients after covid- 19 infection. Egypt Rheumatol Rehabil. (2022) 49:1–8. doi: 10.1186/s43166-022-00165-7 38164791

[40] Berktas BM Gokcek A Hoca NT Koyuncu A . Covid-19 illness and treatment decrease bone mineral density of surviving hospitalized patients. Eur Rev Med Pharmacol Sci. (2022) 26:3046–56. doi: 10.26355/eurrev_202204_28636 35503607

[41] Moga TD Nistor-Cseppento CD Bungau SG Tit DM Sabau AM Behl T . The effects of the 'catabolic crisis' on patients' prolonged immobility after covid-19 infection. Med (Kaunas). (2022) 58:1–15. doi: 10.3390/medicina58060828 35744091 PMC9231342

[42] Al-Azzawi IS Mohammed NS . The impact of angiotensin converting enzyme-2 (ace-2) on bone remodeling marker osteoprotegerin (opg) in post-covid-19 Iraqi patients. Cureus. (2022) 14:e29926. doi: 10.7759/cureus.29926 36348825 PMC9633432

[43] Wang Z Li Z Shen Y Qian S Tang M He J . Long-term effects of covid-19 infection on bone mineral density. J Glob Health. (2024) 14:5029. doi: 10.7189/jogh.14.05029 39421935 PMC11487469

[44] Eastell R O'Neill TW Hofbauer LC Langdahl B Reid IR Gold DT . Postmenopausal osteoporosis. Nat Rev Dis Primers. (2016) 2:16069. doi: 10.1038/nrdp.2016.69 27681935

[45] Khosla S Melton LJ Riggs BL . Clinical review 144: estrogen and the male skeleton. J Clin Endocrinol Metab. (2002) 87:1443–50. doi: 10.1210/jcem.87.4.8417 11932262

[46] Manolagas SC O'Brien CA Almeida M . The role of estrogen and androgen receptors in bone health and disease. Nat Rev Endocrinol. (2013) 9:699–712. doi: 10.1038/nrendo.2013.179 24042328 PMC3971652

[47] LeBlanc A Schneider V Shackelford L West S Oganov V Bakulin A . Bone mineral and lean tissue loss after long duration space flight. J Musculoskelet Neuronal Interact. (2000) 1:157–60. 15758512

[48] Eser P Frotzler A Zehnder Y Wick L Knecht H Denoth J . Relationship between the duration of paralysis and bone structure: a pqct study of spinal cord injured individuals. Bone. (2004) 34:869–80. doi: 10.1016/j.bone.2004.01.001 15121019

[49] Palmisano A Gnasso C Cereda A Vignale D Leone R Nicoletti V . Chest ct opportunistic biomarkers for phenotyping high-risk covid-19 patients: a retrospective multicentre study. Eur Radiol. (2023) 33:7756–68. doi: 10.1007/s00330-023-09702-0 37166497 PMC10173240

[50] Bakhsh N Banjar M Baig M . Correlation of bone density measured on ct chest with the severity of covid-19 infection: a retrospective study. PloS One. (2023) 18:e0286395. doi: 10.1371/journal.pone.0286395 37289783 PMC10249830

[51] Kottlors J Grosse Hokamp N Fervers P Bremm J Fichter F Persigehl T . Early extrapulmonary prognostic features in chest computed tomography in Covid-19 pneumonia: Bone mineral density is a relevant predictor for the clinical outcome - a multicenter feasibility study. Bone. (2021) 144:115790. doi: 10.1016/j.bone.2020.115790 33301962 PMC7720732

[52] Subasi M Duger M Erol C Durur-Subasi A . Opportunistic retrospective assessment of Huac, bone density, Sft and breast density on Ct images and relationship with severity of Covid-19. J Mind Med Sci. (2023) 10:148–155. doi: 10.22543/2392-7674.1371

[53] do Amaral ECA Yokoo P Fonseca E Otoni JC Haiek SL Shoji H . Prognostic factors of worse outcome for hospitalized Covid-19 patients, with emphasis on chest computed tomography data: A retrospective study. Einstein (Sao Paulo). (2022) 20:eAO6953. doi: 10.31744/einstein_journal/2022AO6953 35649055 PMC9126606

[54] Emekli E Bostanci Can EZ . Prognostic value of diaphragm diameter, muscle volume, and bone mineral density in critically ill Covid-19 patients. J Intensive Care Med. (2023) 38:847–55. doi: 10.1177/08850666231169494 37050868 PMC10099913

[55] Del Toro R Palmese F Feletti F Zani G Minguzzi MT Maddaloni E . Relationship between muscle mass, bone density and vascular calcifications in elderly people with Sars-Cov-2 pneumonia. J Clin Med. (2023) 12:1–11. doi: 10.3390/jcm12062372 36983372 PMC10059976

[56] Tahtabasi M Kilicaslan N Akin Y Karaman E Gezer M Icen YK . The prognostic value of vertebral bone density on chest Ct in hospitalized Covid-19 patients. J Clin Densitom. (2021) 24:506–15. doi: 10.1016/j.jocd.2021.07.007 34353732 PMC8302819

[57] Compston J . Glucocorticoid-induced osteoporosis: An update. Endocrine. (2018) 61:7–16. doi: 10.1007/s12020-018-1588-2 29691807 PMC5997116

[58] Buckley L Guyatt G Fink HA Cannon M Grossman J Hansen KE . 2017 American College of Rheumatology guideline for the prevention and treatment of glucocorticoid-induced osteoporosis. Arthritis Rheumatol. (2017) 69:1521–37. doi: 10.1002/art.40137 28585373

[59] Buccino F Zagra L Longo E D'Amico L Banfi G Berto F . Osteoporosis and Covid-19: Detected similarities in bone lacunar-level alterations via combined AI and advanced synchrotron testing. Mater Des. (2023) 231:112087. doi: 10.1016/j.matdes.2023.112087 37323219 PMC10257887

[60] di Filippo L Compagnone N Frara S Allora A Doga M Rovere Querini P . Vertebral fractures at hospitalization predict impaired respiratory function during follow-up of Covid-19 survivors. Endocrine. (2022) 77:392–400. doi: 10.1007/s12020-022-03096-7 35676466

[61] di Filippo L Formenti AM Doga M Pedone E Rovere-Querini P Giustina A . Radiological thoracic vertebral fractures are highly prevalent in Covid-19 and predict disease outcomes. J Clin Endocrinol Metab. (2021) 106:e602–14. doi: 10.1210/clinem/dgaa738 33159451 PMC7797741

[62] Zhang H Balmaceno-Criss M Fruge AM Massey PA Daniels AH Zhang AS . Incidence and outcomes of vertebral compression fracture among patients infected with Covid-19. J Clin Med. (2024) 13:1–13. doi: 10.3390/jcm13247830 39768754 PMC11680045

[63] Patil V Reddy AD Kale A Vadlamudi A Kishore JVS Jani C . Incidental identification of vertebral fragility fractures by chest Ct in Covid-19-infected individuals. Cureus. (2022) 14:e24867. doi: 10.7759/cureus.24867 35698715 PMC9184180

[64] Battisti S Napoli N Pedone C Lombardi M Leanza G Tramontana F . Vertebral fractures and mortality risk in hospitalised patients during the Covid-19 pandemic emergency. Endocrine. (2021) 74:461–9. doi: 10.1007/s12020-021-02872-1 34529239 PMC8444515

[65] Azekawa S Maetani T Chubachi S Asakura T Tanabe N Shiraishi Y . Ct-derived vertebral bone mineral density is a useful biomarker to predict Covid-19 outcome. Bone. (2024) 184:117095. doi: 10.1016/j.bone.2024.117095 38599262

[66] Lui DTW Xiong X Cheung CL Lai FTT Li X Wan EYF . Risks of incident major osteoporotic fractures following Sars-Cov-2 infection among older individuals: A population-based cohort study in Hong Kong. J Bone Miner Res. (2024) 39:551–60. doi: 10.1093/jbmr/zjae041 38477768 PMC11262151

[67] Su SY Sun YF Yeh JJ . Long-term impact of Covid-19 on osteoporosis risk among patients aged ≥50 years with new-onset overweight, obesity, or type 2 diabetes: A multi-institutional retrospective cohort study. Med (Kaunas). (2025) 61:1–12. doi: 10.3390/medicina61081320 40870365 PMC12387685

[68] Alghamdi F Obotiba AD Meertens R Alshalawi O Mokbel K Strain WD . Assessment of bone mineral density, total body composition and joint integrity in long Covid: A 12-month longitudinal feasibility study. J Clin Med. (2025) 14:1–18. doi: 10.3390/jcm14238558 41375862 PMC12692885

[69] Ponzetti M Rucci N . Osteoblast differentiation and signaling: Established concepts and emerging topics. Int J Mol Sci. (2021) 22:1–13. doi: 10.3390/ijms22136651 34206294 PMC8268587

[70] Zhang C Cho K Huang Y Lyons JP Zhou X Sinha K . Inhibition of Wnt signaling by the osteoblast-specific transcription factor Osterix. Proc Natl Acad Sci USA. (2008) 105:6936–41. doi: 10.1073/pnas.0710831105 18458345 PMC2383965

[71] Raucci A Bellosta P Grassi R Basilico C Mansukhani A . Osteoblast proliferation or differentiation is regulated by relative strengths of opposing signaling pathways. J Cell Physiol. (2008) 215:442–51. doi: 10.1002/jcp.21323 17960591

[72] Esen E Lee SY Wice BM Long F . Pth promotes bone anabolism by stimulating aerobic glycolysis via Igf signaling. J Bone Miner Res. (2015) 30:1959–68. doi: 10.1002/jbmr.2556 25990470 PMC4825329

[73] Zoidis E Ghirlanda-Keller C Schmid C . Stimulation of glucose transport in osteoblastic cells by parathyroid hormone and insulin-like growth factor I. Mol Cell Biochem. (2011) 348:33–42. doi: 10.1007/s11010-010-0634-z 21076856

[74] Bar-Shavit Z . The osteoclast: A multinucleated, hematopoietic-origin, bone-resorbing osteoimmune cell. J Cell Biochem. (2007) 102:1130–9. doi: 10.1002/jcb.21553 17955494

[75] Boyle WJ Simonet WS Lacey DL . Osteoclast differentiation and activation. Nature. (2003) 423:337–42. doi: 10.1038/nature01658 12748652

[76] Feng X Teitelbaum SL . Osteoclasts: new insights. Bone Res. (2013) 1:11–26. doi: 10.4248/BR201301003 26273491 PMC4472093

[77] Arai F Miyamoto T Ohneda O Inada T Sudo T Brasel K . Commitment and differentiation of osteoclast precursor cells by the sequential expression of C-Fms and receptor activator of nuclear factor kappaB (Rank) receptors. J Exp Med. (1999) 190:1741–54. doi: 10.1084/jem.190.12.1741 10601350 PMC2195707

[78] Takayanagi H . Osteoimmunology: Shared mechanisms and crosstalk between the immune and bone systems. Nat Rev Immunol. (2007) 7:292–304. doi: 10.1038/nri2062 17380158

[79] Bonewald LF . The amazing osteocyte. J Bone Miner Res. (2011) 26:229–38. doi: 10.1002/jbmr.320 21254230 PMC3179345

[80] Liu Y Zhao L Li M Gong W Wang X Cheng Y . Osteocytes produces Rankl via Wnt-Tgfbeta signaling axis for osteoclastogenesis. Int J Biol Sci. (2025) 21:5821–41. doi: 10.7150/ijbs.117481 41079923 PMC12509901

[81] ten Dijke P Krause C de Gorter DJ Lowik CW van Bezooijen RL . Osteocyte-derived sclerostin inhibits bone formation: Its role in bone morphogenetic protein and Wnt signaling. J Bone Joint Surg Am. (2008) 90:31–5. doi: 10.2106/JBJS.G.01183 18292354

[82] Lacey DL Timms E Tan HL Kelley MJ Dunstan CR Burgess T . Osteoprotegerin ligand is a cytokine that regulates osteoclast differentiation and activation. Cell. (1998) 93:165–76. doi: 10.1016/s0092-8674(00)81569-x 9568710

[83] Yasuda H Shima N Nakagawa N Yamaguchi K Kinosaki M Mochizuki S . Osteoclast differentiation factor is a ligand for osteoprotegerin/osteoclastogenesis-inhibitory factor and is identical to Trance/Rankl. Proc Natl Acad Sci USA. (1998) 95:3597–602. doi: 10.1073/pnas.95.7.3597 9520411 PMC19881

[84] Feng X . Ranking intracellular signaling in osteoclasts. IUBMB Life. (2005) 57:389–95. doi: 10.1080/15216540500137669 16012047

[85] Nardone V D'Asta F Brandi ML . Pharmacological management of osteogenesis. Clinics (Sao Paulo). (2014) 69:438–46. doi: 10.6061/clinics/2014(06)12 24964310 PMC4050321

[86] Hofbauer LC Heufelder AE . Role of receptor activator of nuclear factor-kappaB ligand and osteoprotegerin in bone cell biology. J Mol Med (Berl). (2001) 79:243–53. doi: 10.1007/s001090100226 11485016

[87] Takayanagi H Ogasawara K Hida S Chiba T Murata S Sato K . T-cell-mediated regulation of osteoclastogenesis by signalling cross-talk between Rankl and Ifn-gamma. Nature. (2000) 408:600–5. doi: 10.1038/35046102 11117749

[88] Arron JR Choi Y . Bone versus immune system. Nature. (2000) 408:535–6. doi: 10.1038/35046196 11117729

[89] Sapra L Dar HY Bhardwaj A Pandey A Kumari S Azam Z . Lactobacillus rhamnosus attenuates bone loss and maintains bone health by skewing Treg-Th17 cell balance in Ovx mice. Sci Rep. (2021) 11:1807. doi: 10.1038/s41598-020-80536-2 33469043 PMC7815799

[90] Srivastava RK Sapra L . The rising era of "immunoporosis": Role of immune system in the pathophysiology of osteoporosis. J Inflammation Res. (2022) 15:1667–98. doi: 10.2147/JIR.S351918 35282271 PMC8906861

[91] Hardy R Cooper MS . Bone loss in inflammatory disorders. J Endocrinol. (2009) 201:309–20. doi: 10.1677/JOE-08-0568 19443863

[92] Oliveira TC Gomes MS Gomes AC . The crossroads between infection and bone loss. Microorganisms. (2020) 8:1–18. doi: 10.3390/microorganisms8111765 33182721 PMC7698271

[93] Kazemi-Sufi S Alipour S Rabieepour M Roshan-Milani S Naderi R . Serum proinflammatory cytokines, receptor activator of nuclear factor kappa-beta ligand (Rankl), osteoprotegerin (Opg) and Rankl/Opg ratio in mild and severe Covid-19. BMC Infect Dis. (2024) 24:1047. doi: 10.1186/s12879-024-09941-6 39333916 PMC11428542

[94] Izu Y Mizoguchi F Kawamata A Hayata T Nakamoto T Nakashima K . Angiotensin II type 2 receptor blockade increases bone mass. J Biol Chem. (2009) 284:4857–64. doi: 10.1074/jbc.M807610200 19004830 PMC2742875

[95] Shimizu H Nakagami H Osako MK Hanayama R Kunugiza Y Kizawa T . Angiotensin II accelerates osteoporosis by activating osteoclasts. FASEB J. (2008) 22:2465–75. doi: 10.1096/fj.07-098954 18256306

[96] Sripada L Singh K Lipatova AV Singh A Prajapati P Tomar D . Hsa-mir-4485 regulates mitochondrial functions and inhibits the tumorigenicity of breast cancer cells. J Mol Med (Berl). (2017) 95:641–51. doi: 10.1007/s00109-017-1517-5 28220193

[97] Guo L Wang Q Zhang D . Microrna-4485 ameliorates severe influenza pneumonia via inhibition of the Stat3/Pi3k/Akt signaling pathway. Oncol Lett. (2020) 20:215. doi: 10.3892/ol.2020.12078 32963621 PMC7491079

[98] Chen J Wu X Cai B Su Z Li L An Y . Circulating micrornas as potential biomarkers of HBV infection persistence. Infect Genet Evol. (2017) 54:152–7. doi: 10.1016/j.meegid.2017.06.023 28648686

[99] Sun J Zhuang Z Zheng J Li K Wong RL Liu D . Generation of a broadly useful model for Covid-19 pathogenesis, vaccination, and treatment. Cell. (2020) 182:734–43:e5. doi: 10.1016/j.cell.2020.06.010 32643603 PMC7284240

[100] Haudenschild AK Christiansen BA Orr S Ball EE Weiss CM Liu H . Acute bone loss following Sars-Cov-2 infection in mice. J Orthop Res. (2023) 41:1945–52. doi: 10.1002/jor.25537 36815216 PMC10440245

[101] Awosanya OD Dalloul CE Blosser RJ Dadwal UC Carozza M Boschen K . Osteoclast-mediated bone loss observed in a Covid-19 mouse model. Bone. (2022) 154:116227. doi: 10.1016/j.bone.2021.116227 34607050 PMC8486589

[102] Qiao W Lau HE Xie H Poon VK Chan CC Chu H . Sars-cov-2 infection induces inflammatory bone loss in golden Syrian hamsters. Nat Commun. (2022) 13:2539. doi: 10.1038/s41467-022-30195-w 35534483 PMC9085785

[103] Dette A Moers F Mayr T Stillfried S Bernhardt M Forster S . Differential expression of viral entry protein neuropilin 1 (Nrp1) and neuropilin 2 (Nrp2) in fatal Covid-19. J Virol. (2025) 99:e0138425. doi: 10.1128/jvi.01384-25 41159753 PMC12645918

[104] Delpino MV Quarleri J . Influence of Hiv infection and antiretroviral therapy on bone homeostasis. Front Endocrinol (Lausanne). (2020) 11:502. doi: 10.3389/fendo.2020.00502 32982960 PMC7493215

[105] Hasenmajer V D'Addario NF Bonaventura I Sada V Nardi C Jannini EA . Breaking down bone disease in people living with Hiv: Pathophysiology, diagnosis, and treatment. Adv Exp Med Biol. (2025) 1476:87–110. doi: 10.1007/5584_2024_831 39668274

[106] McGee DM Cotter AG . Hiv and fracture: Risk, assessment and intervention. HIV Med. (2024) 25:511–28. doi: 10.1111/hiv.13596 38087902

[107] Biver E . Osteoporosis and hiv infection. Calcif Tissue Int. (2022) 110:624–40. doi: 10.1007/s00223-022-00946-4 35098324 PMC9013331

[108] SChinas G SChinas I Ntampanlis G Polyzou E Gogos C Akinosoglou K . Bone disease in Hiv: Need for early diagnosis and prevention. Life (Basel). (2024) 14:1–12. doi: 10.3390/life14040522 38672792 PMC11051575

